# Clinicopathological Characteristics of Skin Adnexal Tumors: Insights from a Two-Center Retrospective Study

**DOI:** 10.3390/jcm14144844

**Published:** 2025-07-08

**Authors:** Burcu Sanal Yılmaz, Sibel Acat, Zeliha Esin Çelik

**Affiliations:** 1 Department of Medical Pathology, Faculty of Medicine, Karamanoğlu Mehmetbey University, 70200 Karaman, Turkey; sibel.gungor138@gmail.com; 2 Department of Medical Pathology, Faculty of Medicine, Selçuk University, 42250 Konya, Turkey; dresincelik@hotmail.com

**Keywords:** skin adnexal tumors, histopathology, benign and malignant tumors, tumor size, anatomical localization

## Abstract

**Background/Objectives:** Skin adnexal tumors (SATs) are rare neoplasms originating from sebaceous glands, hair follicles, and sweat glands, often presenting diagnostic challenges due to their histopathological diversity and clinical resemblance to other lesions. This epidemiological and clinicopathological study aimed to evaluate SATs diagnosed between January 2018 and October 2024 across two medical centers in Turkey. **Methods:** A total of 652 cases were analyzed based on demographic features, tumor size, anatomical localization, and histological subtypes per the 2018 WHO classification. The study also explored the predictors of malignancy, including tumor size and multifocality. **Results:** Among the cases, 98% were benign and 2% malignant. Sebaceous tumors were the most common (34.5%), followed by eccrine/apocrine (34.2%) and follicular tumors (31.3%). Benign tumors showed a slight female predominance (56.6%), while malignant tumors were more frequent in males (61.5%). The majority of tumors were located in the head and neck region (84.6%), and a tumor size >20 mm was significantly associated with malignancy. **Conclusions**: This study, one of the largest series from Turkey, highlights the importance of clinicopathological correlation in SATs. It contributes to the literature by identifying size-based cut-off values for malignancy prediction and by assessing interobserver agreement, multifocality, and tumor subtype distribution.

## 1. Introduction

Skin adnexal tumors (SATs) are a group of neoplasms that originate from adnexal structures, such as sebaceous glands, hair follicles, and sweat glands, and exhibit unique heterogeneous features. These tumors are presented with varied clinical signs; they may range from asymptomatic nodules to more complex lesions that can be challenging to diagnose by clinicians and pathologists [1,2,3]. Encompassing both malignant and benign forms of SATs, the former are often aggressive in nature and require quick and accurate diagnosis [4].

The classification of SATs has come a long way since the introduction of the 2018 World Health Organization (WHO) classification, which helped in making a complete framework for diagnosis (WHO Classification of Skin Tumors, 5th Edition). According to this, SATs are “appendageal tumors” and are further split into the following four categories based on their differentiation: apocrine and eccrine differentiation, follicular differentiation, sebaceous differentiation, and region-specific appendageal tumors [5]. Efficient diagnosis often involves a combination of clinical assessment, histopathological examination, and specialized diagnostic approaches, like immunohistochemistry and molecular techniques [6].

According to some studies, SATs are considered rare with them attributing to less than five percent of known skin tumors [7]. However, their occurrence is expected to be different due to the population as well as healthcare settings. For example, research conducted in Iran showed a prevalence of 3.3% among 30,000 pathology records [8]. Likewise, Paraguay and Nigeria have reported a prevalence of 1.4% and 0.9%, respectively, over long periods [8]. In Pakistan, SATs of benign nature were found to be more, while the sweat gland tumors had the upper hand (62%) and were followed by hair follicle tumors (28.5%) and sebaceous tumors (9.5%) [8,9]. These differences illustrate the geographic, genetic, and demographic factors that “determine” the distribution and prevalence of SATs.

Skin adnexal tumors (SATs), although rare, are clinically significant due to their association with syndromic conditions such as Muir–Torre syndrome. The early identification of these associations is critical for timely treatment and genetic counseling [1,8]. Adnexal neoplasms may serve as cutaneous markers for a wide range of hereditary syndromes, making accurate diagnosis essential to alert clinicians to such possibilities. Beyond hereditary syndromes, recent studies have identified genetic alterations in sporadic SATs; although, their etiology and pathogenesis remain poorly understood [10,11]. Furthermore, many patients affected by syndromic SATs may present with additional systemic findings, underscoring the need for a multidisciplinary approach to care [12].

Demographic and geographic factors are a crucial determinant in the distribution of SATs. South Asian populations typically have a higher relative frequency of benign SATs with eccrine differentiation, while their Western counterparts have a higher frequency of benign SATs with sebaceous gland differentiation [8,9]. Moreover, Turkey’s data correspond to this data, where it is shown that SATs are mostly benign (95.4%) and are commonly found in the head and neck regions (66.1%) [3].

The literature has underscored the difficulties in combining clinical and histopathological diagnoses in SATs and has stressed the importance of particular consideration to histological diagnosis [3,12]. At the same time, however, the progress in molecular pathology concerning SATs offers new opportunities concerning its pathogenesis and its prognostic factors, as well as target interventions [6].

The aim of this study was to evaluate the epidemiological and histopathological characteristics of skin adnexal tumors in a large series of 652 cases diagnosed at two major medical centers in Turkey. Using the 2018 WHO classification, the study investigated subtype distribution and explored associations between tumor size, age, localization, multifocality, and malignancy. In addition, a receiver operating characteristic (ROC) analysis was performed to determine tumor size cut-off values predictive of malignancy. Interobserver agreement among pathologists was also assessed to evaluate diagnostic consistency.

This study fills a gap in the literature by providing one of the most comprehensive datasets on SATs in Turkey. Its contributions include the analysis of a large multicenter cohort, the identification of malignancy-associated cut-off values for tumor size, data on multifocality patterns, and interobserver agreement among pathologists. By strengthening the regional evidence base, the findings are expected to support earlier recognition and accurate classification of SATs, including rare malignant subtypes.

## 2. Materials and Methods

This retrospective study was carried out in two medical centers, namely, the Training and Research Hospital, Department of Medical Pathology and the University Faculty of Medicine, Department of Medical Pathology. A total of 652 cases of skin adnexal tumors (SATs) were diagnosed between January 2016 and October 2024. Only cases that had available tissue blocks and slides and complete clinical and demographic information were selected to ensure data integrity and reliability. Ethics committee approval for this study was obtained from the Ethics Committee of Karamanoğlu Mehmetbey University Faculty of Medicine (Ethics Committee Approval Number: E-11095095-050.04-222463). The study was conducted in accordance with the ethical principles of the Declaration of Helsinki.

Inclusion and Exclusion Criteria
Inclusion Criteria:

1. Cases reported as SATs following histopathological examination.
2. Paraffin-embedded blocks and corresponding Hematoxylin and Eosin (H&E)-stained slides must be available for review.
3. All demographic and clinical data, such as age, gender, the localization of the lesion, and the size of the tumor, must be provided.

Exclusion Criteria:

Cases were excluded from the study if any essential demographic information—particularly tumor size or anatomical location—was missing. Additionally, cases in which a benign or malignant distinction could not be made upon histopathological evaluation, or where tissue slides or paraffin blocks were unavailable, were also excluded. For the subgroup analyses involving immunohistochemical data, cases that lacked staining with the relevant antibodies or had inadequate staining quality were not included in the evaluation.

Data Collection

Patient records were systematically reviewed to extract data on age, gender, lesion size, anatomical localization, the number of lesions, and histopathological diagnosis. The classification of SATs into benign or malignant categories and their histological subtypes was based on the 2018 WHO classification of skin tumors. The localization of tumors was categorized into the head and neck region, trunk, upper extremities, lower extremities, and other regions.

All H&E-stained slides were reexamined by three experienced pathologists independently. Discrepancies in the diagnosis were resolved through consensus discussions. Immunohistochemical stains, if previously performed, were also reviewed to corroborate the histological findings. Tumor size was measured in millimeters based on the maximum diameter noted during pathological examination.

Interobserver Agreement

To assess interobserver reliability, all histological classifications were independently performed by three pathologists. The level of agreement between the observers was evaluated using Cohen’s Kappa coefficient. The kappa values indicated excellent agreement between all pairs of observers, as follows:

- Pathologist 1 vs. 2: κ = 0.915, *p* < 0.001
- Pathologist 1 vs. 3: κ = 0.959, *p* < 0.001
- Pathologist 2 vs. 3: κ = 0.867, *p* < 0.001

These results demonstrated a high level of classification consistency and supported the reliability of the histopathological assessments.

Statistical Analysis

The data were analyzed using the SPSS 25.0 software package to derive meaningful insights. Descriptive statistics were used to summarize variables such as age, gender, tumor size, anatomical localization using frequency (*n*), percentage (%), arithmetic mean (X), standard deviation (SD), and median (min–max). The comparative analysis of categorical variables, including benign versus malignant cases and gender distribution, was performed using the Chi-square test, with Fisher’s exact test applied when more than 20% of cells in contingency tables had expected values less than 5. Statistical significance was defined as *p* < 0.05 for all tests. The distribution of SATs was evaluated by stratifying data into age groups (≤20 years, 21–40 years, 41–60 years, ≥61 years) and gender. Additionally, tumor size was analyzed by grouping measurements into ≤10 mm, 11–20 mm, 21–30 mm, and >30 mm, examining their correlation with benign or malignant status.

Quality Control

Every pathological diagnosis was confirmed by re-examining the histology slides in order to guarantee the validity of the information. Two separate investigators inputted the data into the clinicopathological database in order to reduce the risks of error, and routine checks were performed to ensure consistency.

This framework in method allows for complete and thorough analysis of SATs, as well as the characteristics and variety of their clinicopathological features around the world by different demographic and anatomical groups.

## 3. Results

This study analyzed 652 cases of skin adnexal tumors (SATs), providing a detailed evaluation of their histopathological subtypes, anatomical localization, gender distribution, age patterns, and tumor size. Among these cases, 92.7% (*n* = 639) were classified as benign, while 7.3% (*n* = 13) were malignant.

Multiple lesions were observed in 7% (*n* = 46) of the cases. According to the updated data (Table 1), the most frequent pattern was solitary lesions (92.94%, *n* = 606), followed by cases with two lesions (4.14%, *n* = 27) and three lesions (1.69%, *n* = 11). Cases with more than three lesions were rare.

Eccrine/apocrine tumors accounted for 34.2% (*n* = 223) of all SATs, with syringoma being the most common subtype, representing 30.0% (*n* = 67) of these tumors. Other subtypes included hidrocystoma (26.0%, *n* = 58), hidradenoma (10.8%, *n* = 24), poroma (9.4%, *n* = 21), and spiradenoma (7.2%, *n* = 16). Malignant eccrine/apocrine tumors, such as hidradenocarcinoma and porocarcinoma, were rare but clinically significant, comprising less than 5% of this category. Follicular tumors constituted 31.3% (*n* = 204) of SATs, with pilomatricoma being the most frequent subtype, accounting for 52.5% (*n* = 107) of cases. Other notable subtypes included trichoblastoma (25.0%, *n* = 51) and proliferating trichilemmal tumor (10.3%, *n* = 21). Malignant forms, such as trichoblastic carcinoma and trichilemmal carcinoma, were rare and accounted for less than 3%. Sebaceous tumors represented the largest group, comprising 34.5% (*n* = 225) of all SATs. Sebaceous hyperplasia was the most prevalent subtype, accounting for 62.2% (*n* = 140), followed by sebaceous nevus (28.4%, *n* = 64). Malignant sebaceous carcinoma was rare (1.7%, *n* = 4) but significant due to its aggressive nature and potential syndromic associations (Table 2).

A slight female predominance was observed, with 56.6% (*n* = 369) of SATs occurring in women and 43.4% (*n* = 283) in men. Subtype-specific analysis revealed that syringoma was significantly more frequent in females, accounting for 39.9% of eccrine/apocrine tumors in this group, compared to 14.1% in males. In contrast, subtypes like hidrocystoma and poroma were more commonly observed in males. Pilomatricoma, the most common follicular tumor, showed a balanced distribution but with a slight female predominance (56.0% in females vs. 48.4% in males). Similarly, sebaceous hyperplasia exhibited nearly equal gender distribution, with a prevalence of 63.2% in females and 61.1% in males.

The head and neck region was the predominant site for SATs, accounting for 84.6% (*n* = 551) of cases. Among these, the face was the most frequently affected subregion, comprising 70.8% (*n* = 461) of cases, followed by the scalp (9.4%, *n* = 61), neck (4.2%, *n* = 27), and ear (0.3%, *n* = 2). Other anatomical localizations included the upper extremities (6.7%, *n* = 44), trunk (3.5%, *n* = 23), lower extremities (3.1%, *n* = 20), and genital area (2.1%, *n* = 14) (Table 3). These results underscore the preference of SATs for the head and neck region, particularly the face, with lesions in other regions being relatively uncommon.

Tumor size analysis revealed that benign lesions were predominantly ≤9.5 mm in diameter, accounting for 60% (*n* = 393) of cases, with common subtypes including sebaceous hyperplasia, syringoma, and pilomatricoma. Lesions measuring 9.51–15.50 mm comprised 22% (*n* = 142) of cases and included larger benign subtypes, such as sebaceous nevus, as well as some malignant lesions. Tumors measuring 15.51–25.50 mm accounted for 12% (*n* = 81), primarily consisting of malignant subtypes like porocarcinoma and hidradenocarcinoma. Advanced malignant tumors exceeding 25.51 mm were rare, representing 6% (*n* = 36) of cases (Table 4). This observation indicates that lesions of larger size are more likely to be malignant; however, small-sized malignant lesions may be missed when these size thresholds are taken as strict diagnostic criteria.

**Figure 1:**
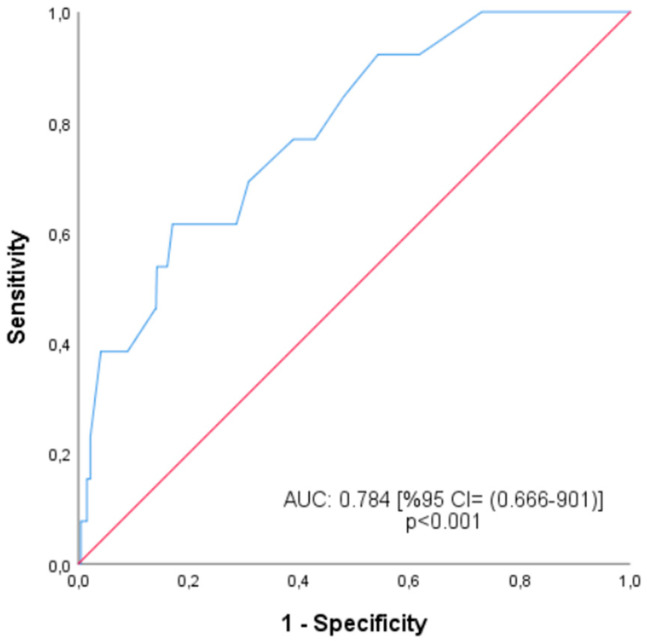
ROC curve for tumor size.

Receiver operating characteristic (ROC) analysis was performed to evaluate the diagnostic performance of tumor diameter in distinguishing between benign and malignant SATs (Figure 1). The area under the curve (AUC) was calculated as 0.784 (95% CI: 0.666–0.901; *p* < 0.001), indicating a moderate discriminatory power. The optimal cutoff point was determined to be 9.5 mm, with a sensitivity of 76.9%, specificity of 61.0%, and a positive likelihood ratio (LR^+^) of 2.0. Tumors larger than this cutoff were significantly associated with malignancy (*p* < 0.05).

**Figure 2:**
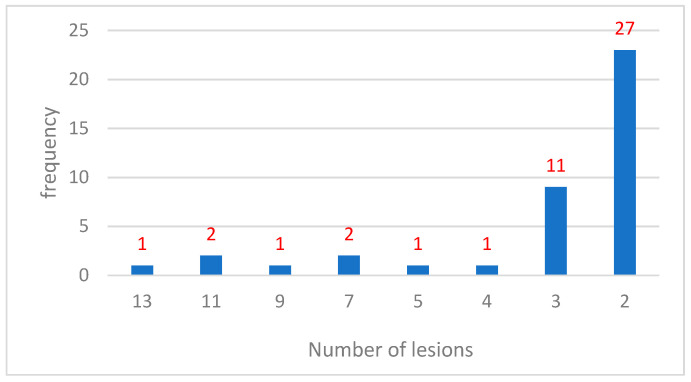
Multiples.

Age played a pivotal role in the distribution of SATs, with the majority of lesions (62%) occurring in the 30–60-year age group. Pilomatricoma was predominantly observed in younger individuals (≤20 years), accounting for 52 cases. Syringoma and sebaceous hyperplasia were most common in the 41–60-year age group, while malignant lesions, including sebaceous carcinoma and hidradenocarcinoma, were primarily observed in patients aged ≥50 years (Figure 2). These findings underscore the significance of age as a diagnostic parameter, with younger age groups being associated with benign subtypes and older age groups showing a higher prevalence of malignancies (Table 5).

**Figure 3:**
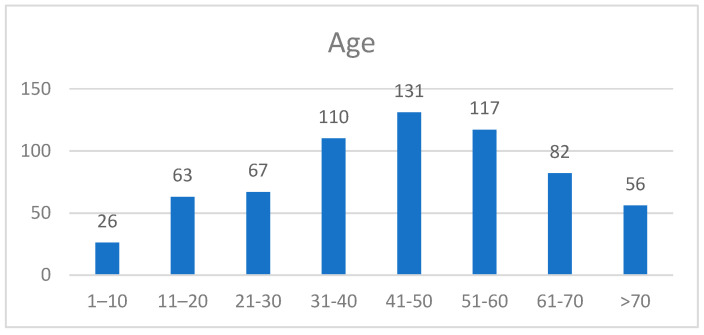
Distribution of age ranges.

**Figure 4:**
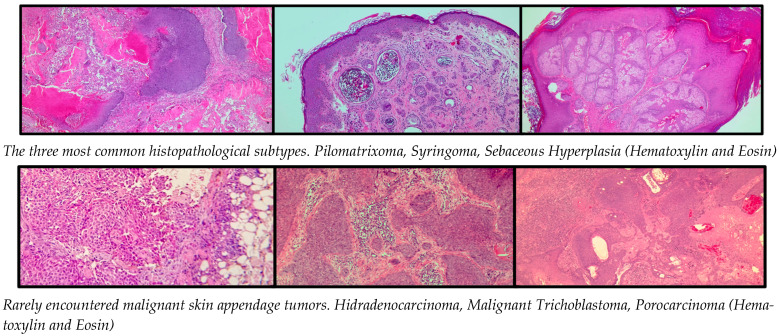
Histopathological appearance of a cutaneous adnexal tumor.

Multiple lesions were identified in 7% (*n* = 46) of cases and were predominantly benign. Syringoma exhibited the highest frequency of multiple lesions, often localized to the periorbital region and predominantly observed in females. Sebaceous hyperplasia frequently appeared as multiple papules in sun-exposed areas, particularly in the head and neck region (Figure 3). Pilomatricoma occasionally presented as clustered lesions (Figure 4), typically in younger patients and localized to the upper extremities and face. Multiple malignant lesions were exceedingly rare.

**Figure 5:**
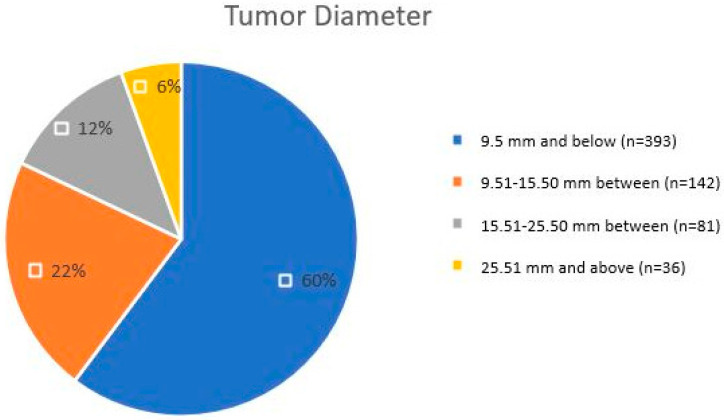
Distribution according to tumor diameters.

Among the 652 cases analyzed, 98.0% (*n* = 639) were benign, and 2.0% (*n* = 13) were malignant (Figure 5). Eccrine/apocrine tumors exhibited the highest malignancy rate (3.1%), followed by follicular tumors (2.5%) and sebaceous tumors (1.7%). Malignant lesions were more frequently observed in males (61.5%) and in patients aged ≥50 years. Tumor size was a significant indicator of malignancy, with malignant lesions generally exceeding 15 mm in diameter compared to benign lesions, which were predominantly ≤9.5 mm. These findings highlight the predominantly benign nature of SATs, while emphasizing the need for careful evaluation of larger lesions in older patients to identify rare malignant subtypes.

## 4. Discussion

Adnexal cutaneous tumors are, for the most part, non-threatening skin growths. However, their ability to morphologically change greatly makes diagnosis problematic because of the similarity with malignant tumors like basal cell and squamous cell carcinomas [8]. The diagnosis of SATs is particularly difficult owing to their overlap in morphology between benign and malignant subtypes. They are also diverse in nature. This requires that a thorough histopathological subtyping be carried out for effective clinical management and prognosis [9]. Additionally, studies indicate that genetic factors, environmental factors, or population factors influences tumor behavior and distribution [3,8]. This study reiterates the predominance of benign SATs, as 98% of the cases in our cohort are benign subtypes, while only 2% are malignant. Some rare malignant types, like sebaceous carcinoma, porocarcinoma, and spiradenocarcinoma, were found, as also noted by Oyasiji et al., underscoring the need for early diagnosis and treatment in these aggressive forms [4].

The distribution of SATs in this study corresponds with other parts of the world. For example, an Iranian study showed that 93.8% of these tumors are benign, while 6.2% are malignant with sebaceous carcinoma being the most common [8]. Similarly, studies from South India and Nigeria also demonstrated high rates of benign tumors, with pilomatricoma, sebaceous hyperplasia, and syringoma being the most common subtypes [13,14]. These findings highlight the global predominance of benign SATs and underscore the rarity of malignancies. However, the presence of rare but aggressive malignant subtypes like sebaceous carcinoma requires careful histopathological evaluation and awareness among clinicians. Likewise, a Turkish study by Aslan et al. [3] found that the benign rate is 95.4% as opposed to a malignant rate of 4.6%, which is also close to our results. On the other hand, Fulton, Kaley, and Gardner [1] reported having more difficulty in diagnosis due to the similarity in traits displayed by the benign type and malignant forms, thus showing the importance of histopathological examination and the need for evaluation by experts.

Our research showed that benign SATs are more predominant in females (56.6%), which echoes a number of local studies including those carried out in Pakistan and India [9,12]. A balanced gender distribution was reported in some studies, such as an Iranian cohort where 51% of cases occurred in females and 49% in males [8]. This variability suggests that gender-based differences in SAT prevalence may be influenced by regional and population-specific factors. For instance, sebaceous hyperplasia has shown a nearly equal distribution, while pilomatricoma and syringoma exhibit a slight female predominance, as noted in our study and others [9,15]. The malignant subtypes, however, were found to have slight male predominance like the findings by Wada et al. [16] who reported an increased prevalence of malignant SATs among the males above 50 years. The age-specific trends in this study did support the earlier reports with younger individuals having more pilomatricoma and older adults exhibiting more malignant lesions. This age-related pattern reflects the biology of SATs, where benign subtypes like pilomatricoma are more common in younger age groups, while malignancies tend to arise in older individuals, consistent with studies from India and Iran [8,17].

The head and neck region remains the most common location for SATs and has a predilection of occurrence of 84.6% which is in agreement with earlier studies, including Kamyab-Hesari et al. [8] and Bhat et al. [12]. Notably, sebaceous tumors and syringomas were predominantly localized to the face, aligning with findings from Turkey, where sebaceous hyperplasia was frequently observed in the head and neck region [3]. A pediatric study further demonstrated a higher predilection for this region in children, where 87% of SATs were found in the head and neck, underscoring the consistency of anatomical distribution across age groups [18]. Differences in localization, such as a higher involvement of extremities in studies from Pakistan, may reflect genetic or environmental factors unique to those populations [9]. The increased occurrence of sebaceous hyperplasia and pilomatricoma in this region also makes sense with the anatomical preferences for benign types of tumors. Even studies by Gibbs, Yeung, and Blalock [7] pointed to this being due to environmental and genetic factors, so this suggest the need for more research. Our findings are largely in agreement with previous large cohort studies from Turkey, South Asia, and the Middle East. However, unlike Kamyab-Hesari et al.’s study involving over 1000 cases, our series includes detailed histological stratification along with immunohistochemical support in selected cases, which allowed better subtyping. While most studies focused primarily on general frequencies, our data emphasize specific age-, sex-, and location-based patterns supported by quantitative proportions. For example, sebaceous carcinoma was slightly more frequent in our cohort (1.7%) than in the Pakistani series by Saleem et al. (1.1%) [9], possibly reflecting referral bias or regional genetic variation. Similarly, pilomatricoma predominance (52.5% of follicular tumors) in younger patients was more marked than that reported in Indian datasets, such as Kaur et al. [10], suggesting subtle but meaningful demographic or geographic differences.

A significant discriminator factor between benign and malignant lesions is tumor size. Tumors smaller than 10 mm tend to be benign, while tumors larger than 20 mm tend to be malignant lesions. In our cohort, 75% of benign lesions measured ≤10 mm, which is consistent with reports from Turkey and South India, where benign SATs were predominantly small in size [3,13]. Larger lesions, particularly those exceeding 30 mm, were exclusively malignant, a finding that reinforces the diagnostic value of tumor size in distinguishing benign and malignant subtypes. Similar correlations between tumor size and malignancy have been observed in studies from Iran and South Asia [8,17]. This has also been noted by Plotzke, Adams, and Harms [6]. These findings highlight the need to factor in the size of the tumor when constructing diagnostic algorithms, especially for lesions that have clinical pictures that are not specific. To support this, ROC analysis in our study demonstrated an AUC of 0.784 (95% CI: 0.666–0.901; *p* < 0.001), indicating moderate diagnostic power for tumor diameter in predicting malignancy. The optimal cut-off value was 9.5 mm, yielding a sensitivity of 76.9% and specificity of 61.0%. Importantly, specificity increased with tumor size, peaking at 95.1% for lesions ≥25.5 mm, underlining its clinical relevance. Based on this stratification, tumors ≤9.5 mm were predominantly benign (60%), whereas those ≥25.5 mm carried a significantly higher risk of malignancy. These data suggest that tumor size should be considered a practical criterion in risk stratification algorithms, especially when combined with anatomical location and patient age. We recommend heightened suspicion for malignancy in SATs larger than 25 mm, especially those located outside the head and neck region or arising in older adults. A structured clinical decision-making algorithm incorporating size thresholds—such as tumors >15 mm suggesting moderate risk and tumors ≥25 mm indicating high risk of malignancy—may improve the early identification of aggressive subtypes. Combining tumor diameter with patient age and anatomical location could help prioritize cases for urgent histopathologic evaluation.

Eccrine/apocrine tumors constituted 34.2 percent of all cases, with the most common subtype being syringoma followed by hidrocystoma. Similar findings were reported by Kazakov et al. [5] and Kamyab-Hesari et al. [8] from South Asian and Middle Eastern countries who noted similar distributions. Sebaceous tumors, which had the highest number in our cohort (34.5%), were mostly benign, for example, sebaceous hyperplasia accounted for 62.2% of cases. This is in agreement with data from Pakistan and Turkey where sebaceous tumors were noted as the most common subtype as well [3,9]. On the other hand, malignant sebaceous carcinoma, although rare compared to other tumors, is clinically important because of relevant syndromic associations such as Muir–Torre syndrome [2].

The histopathologic assessment within this study revealed the actual relevance of accurate subtyping to direct management decisions. For instance, pilomatricoma comprising 52.5% of follicular tumors showed a strong preference for younger patients as compared to truly malignant subtypes, such as trichoblastic carcinoma, which were extremely rare. Such results are consistent with worldwide trends and point toward the value of histopathology skill in diagnosing benign versus malignant forms [3,13]. In conclusion, our study highlights the predominantly benign nature of SATs and underscores the importance of histopathological evaluation for accurate diagnosis and effective management. While benign subtypes, such as sebaceous hyperplasia, pilomatricoma, and syringoma, dominate the clinical landscape, the presence of rare but aggressive malignancies necessitates heightened vigilance, particularly in older populations.

### Strengths and Limitations

This study has several limitations inherent to its retrospective design. Most notably, selection bias may have occurred due to the exclusion of cases with incomplete demographic or histopathological data and missing pathological materials. Additionally, the study was conducted at only two centers, which may limit the generalizability of the findings to broader populations.

Although our study focused primarily on histopathological subtyping, recent literature emphasizes the role of molecular and genetic profiling in the diagnosis and understanding of adnexal tumors. According to the WHO 5th edition, some SATs may serve as cutaneous indicators of hereditary syndromes, such as Muir–Torre and Cowden syndromes, making accurate diagnosis essential for genetic counseling. Furthermore, emerging studies have identified alterations in genes like *TP53, CYLD, HRAS,* and *BRAF* in sporadic adnexal tumors [6,10,11]. Future research incorporating molecular markers and immunohistochemical panels may enhance diagnostic precision and enable tailored treatment strategies.

## 5. Conclusions

The results support international benchmarks by showing the common occurrence of sebaceous and eccrine/apocrine tumors with a tendency for the head and neck region. Also, upon thorough examination of the tumors, other important indicators like patient age and location of the lesion proved crucial for distinguishing between benign and malignant lesions.

In particular, diagnostic challenges around SATs are central to the study. The importance of a solid pathologic examination and certain immunohistochemical and molecular approaches to obtain the right single diagnosis is duly addressed. These results are important for doctors and pathologists practicing in areas with scarce information on SATs for effective diagnosis and management.

More attention should be directed toward using molecular pathology to improve the mechanisms of SAT pathogenesis, as well as larger, multicenter databases to see if the results are similar in different populations. This will aid in formulating better algorithms for the diagnosis and management of SATs and result in better patient care.

## Figures and Tables

**Table 1:** Frequency of lesions.

Number of Lesions (Number)	*n*	%
13	1	0.15
11	2	0.31
9	1	0.15
7	2	0.31
5	1	0.15
4	1	0.15
3	11	1.69
2	27	4.14
1	606	92.94

**Table 2:** Distribution of eccrine/apocrine, follicular, and sebaceous tumors.

		Count	Column N %
Eccrine/Apocrine Tumor	Syringoma	67	30.0%
	Hidrocystoma	58	26.0%
	Hidradenoma	24	10.8%
	Poroma	21	9.4%
	Spiradenoma	16	7.2%
	Benign mixed tumor	11	4.9%
	Hidradenoma papilliferum	8	3.6%
	Syringocystadenoma papilliferum	6	2.7%
	Poroid hidradenoma	4	1.7%
	Hidradenocarcinoma	3	1.3%
	Porocarcinoma	3	1.3%
	Cylindroma	1	0.4%
	Spiradenocarcinoma	1	0.4%
Follicular Tumor	Pilomatrixoma	107	52.5%
	Trichoblastoma	51	25.0%
	Proliferating trichilemmal tumor	21	10.3%
	Trichofolliculoma	11	5.4%
	Folliculosebaceous cystic hamartoma	5	2.5%
	Winer’s dilated pore	5	2.5%
	Malignant trichoblastoma	1	0.5%
	Trichilemmal carcinoma	1	0.5%
	Trichilemmoma	1	0.5%
	Trichoepithelioma	1	0.5%
Sebaceous Tumor	Sebaceous hyperplasia	140	62.2%
	Sebaceous nevus	64	28.4%
	Sebaceoma	9	4.0%
	Sebaceous adenoma	8	3.6%
	Sebaceous carcinoma	4	1.7%

*This table presents the distribution and percentage of various types of eccrine/apocrine, follicular, and sebaceous tumors observed in the study. Each tumor type is categorized with its frequency (Count) and proportion of the total cases (Column N %) within its respective group.*

**Table 3:** Distribution of eccrine/apocrine, follicular, and sebaceous tumors by gender.

		Gender			
		Male		Female	
		Count	Column N %	Count	Column N %
Eccrine/Apocrine Tumors	Hidrocystoma	25	29.4%	33	23.9%
	Hidradenoma	12	14.1%	12	8.7%
	Poroma	12	14.1%	9	6.5%
	Syringoma	12	14.1%	55	39.9%
	Benign Mixed Tumor	8	9.4%	3	2.2%
	Spiradenoma	8	9.4%	8	5.8%
	Syringocystadenoma Papilliferum	2	2.4%	4	2.9%
	Poroid Hidradenoma	3	3.5%	1	0.7%
	Hidradenocarcinoma	1	1.2%	2	1.4%
	Porocarcinoma	1	1.2%	2	1.4%
	Spiradenocarcinoma	1	1.2%	0	0.0%
	Hidradenoma Papilliferum	0	0.0%	8	5.8%
	Cylindroma	0	0.0%	1	0.7%
Follicular Tumors	Pilomatrixoma	46	48.4%	61	56.0%
	Trichoblastoma	22	23.2%	29	26.6%
	Proliferating Trichilemmal Tumor	9	9.5%	12	11.0%
	Trichofolliculoma	9	9.5%	2	1.8%
	Folliculosebaceous Cystic Hamartoma	3	3.2%	2	1.8%
	Winer’s Dilated Pore	3	3.2%	2	1.8%
	Trichilemmal Carcinoma	1	1.1%	0	0.0%
	Trichilemmoma	1	1.1%	0	0.0%
	Trichoepithelioma	1	1.1%	0	0.0%
	Malignant Trichoblastoma	0	0.0%	1	0.9%
Sebaceous Tumors	Sebaceous Hyperplasia	63	61.1%	77	63.2%
	Sebaceous Nevus	27	26.2%	37	30.3%
	Sebaceous Adenoma	5	4.9%	3	2.5%
	Sebaceoma	4	3.9%	5	4.1%
	Sebaceous Carcinoma	4	3.9%	0	0.0%

*This table shows the distribution and percentages of eccrine/apocrine, follicular, and sebaceous tumors by gender (male and female). Each tumor type is presented with the number of cases (Count) and the percentage (Column N %) observed in male and female groups.*

**Table 4:** Distribution of eccrine/apocrine, follicular, and sebaceous tumors by age groups.

		1–10	11–20	21–30	31–40	41–50	51–60	61–70	>70	Total
**Eccrine/Apocrine Tumors**	Syringoma	0	0	0	16	24	23	4	0	67
	Hidrocystoma	1	1	3	8	19	13	9	4	58
	Hidradenoma	0	0	3	3	4	6	3	5	24
	Poroma	0	1	1	2	3	5	3	6	21
	Spiradenoma	0	0	1	3	2	3	5	2	16
	Benign mixed tumor	0	0	2	3	0	2	3	1	11
	Hidradenoma papilliferum	0	0	0	2	3	3	0	0	8
	Syringocystadenoma papilliferum	0	1	0	3	2	0	0	0	6
	Poroid hidradenoma	0	1	0	0	1	2	0	0	4
	Hidradenocarcinoma	0	0	0	0	0	1	0	2	3
	Porocarcinoma	0	0	0	0	0	0	0	3	3
	Cylindroma	0	0	0	0	0	0	1	0	1
	Spiradenocarcinoma	0	0	0	0	0	0	1	0	1
**Follicular Tumors**	Pilomatrixoma	21	31	20	16	8	5	3	3	107
	Trichoblastoma	1	2	8	7	8	10	9	6	51
	Proliferating trichilemmal tumor	0	1	2	2	1	5	7	3	21
	Trichofolliculoma	0	0	1	2	1	2	4	1	11
	Folliculosebaceous cystic hamartoma	0	0	3	0	1	0	1	0	5
	Winer’s dilated pore	0	0	1	0	2	1	1	0	5
	Malignant trichoblastoma	0	0	0	0	0	1	0	0	1
	Trichilemmal carcinoma	0	0	0	0	0	0	0	1	1
	Trichilemmoma	0	0	0	0	0	0	0	1	1
	Trichoepithelioma	0	0	0	0	0	1	0	0	1
**Sebaceous Tumors**	Sebaceous hyperplasia	1	12	15	24	35	28	17	8	140
	Sebaceous nevus	2	12	7	17	14	5	6	1	64
	Sebaceoma	0	0	0	2	2	0	3	2	9
	Sebaceous adenoma	0	1	0	0	1	1	2	4	9
	Sebaceous carcinoma	0	0	0	0	0	0	1	3	4

*This table presents the numerical distribution of eccrine/apocrine, follicular, and sebaceous tumors across different age groups. Each tumor type is displayed with the number of cases observed in each age range, along with the total number of cases.*

**Table 5:** Distribution of tumors by gender and anatomical regions.

		Gender		
		M	F	Total
**Head and Neck**	*n* (% of Total)	237 (36.3)	314(48.3)	551 (84.6)
Face	*n* (% of Total)	192 (29.4)	269 (41.3)	461 (70.8)
Neck	*n* (% of Total)	14 (2.1)	13 (2.0)	27 (4.2)
Ear	*n* (% of Total)	1 (0.2)	1 (0.2)	2 (0.3)
Scalp	*n* (% of Total)	30 (4.6)	31 (4.8)	61 (9.4)
Upper Extremity	*n* (% of Total)	21 (3.2)	23 (3.5)	44 (6.7)
Lower Extremity	*n* (% of Total)	9 (1.4)	11 (1.7)	20 (3.1)
Trunk	*n* (% of Total)	13 (2.0)	10 (1.5)	23 (3.5)
Genital Region	*n* (% of Total)	3 (0.5)	11 (1.7)	14 (2.1)
Benign	*n* (% of Total)	275 (42.2)	364 (55.8)	639 (98.0)
Malignant	*n* (% of Total)	8 (1.2)	5 (0.8)	13 (2.0)
Total	*n* (% of Total)	**283** (43.4)	**369** (56.6)	**652** (100.0)

*This table shows the numerical and percentage distribution of benign and malignant tumors by gender (male and female) and anatomical regions. For each anatomical region and tumor type, the total number of cases along with the proportions in male and female groups are presented.*

## Data Availability

The data presented in this study are available upon reasonable request from the corresponding author.
